# Health literacy and medication adherence in psoriasis patients: a survey in Iran

**DOI:** 10.1186/s12875-022-01719-6

**Published:** 2022-05-10

**Authors:** Yasaman Avazeh, Soheila Rezaei, Peivand Bastani, Gholamhossein Mehralian

**Affiliations:** 1 Department of Pharmacoeconomics and Pharma Management, School of Pharmacy, Tehran University of Medical Sciences, Tehran, Iran; 2 Department of Pharmacoeconomics and Pharma Management, School of Pharmacy, Shahid Beheshti University of Medical Sciences, Tehran, Iran; 3 Research Fellow, Faculty of Health and Behavioral Sciences, School of Dentistry, University of Queensland, Brisbane, QLD 4072 Australia; 4 Nottingham Business School, Nottingham Trent University, Nottingham, UK; 5 Shahid Beheshti University of Medical Sciences, Tehran, Iran

**Keywords:** Medication, Adherence, Health literacy, Psoriasis

## Abstract

**Background:**

Medication adherence among Psoriasis patients is often inadequate identified as a significant problem in Psoriasis symptoms management. Poor medication adherence could necessitate stronger and more expensive medications, which could place a significant burden on the healthcare system. Moreover, the importance of health literacy assessment as a factor influencing adherence in psoriasis patients cannot be overstated. This study aimed to evaluate the medication adherence level of Iranian Psoriasis patients and its relationship with the patients’ health literacy level and demographic conditions.

**Methods:**

This is a cross-sectional study among Iranian psoriasis patients conducted through a web-based questionnaire survey between 26 July 2020 and 5 January 2021 and a total of 575 samples were collected. The questionnaire consisted of 3 sections: First, demographic information and disease characteristics were evaluated. Second, the medication adherence was evaluated by using valid Morisky Medication Adherence Scale-8 (MMAS-8), and, finally, the health literacy was evaluated by using Health Literacy for Iranian Adults (HELIA). Data were analyzed using SPSS software, version 22 with descriptive statistics; Chi-square and Kruskal–Wallis tests. Stepwise multiple linear regression was also used to evaluate the impact of independent variables related on medication adherence score.

**Results:**

Results showed that the mean health literacy score in the study population was 74.3 ± 14.23, and the mean medication adherence score was 4.1 ± 2.18. Out of the total participants, 28.8% had high health literacy, 67.1% had adequate health literacy, and 4% had inadequate health literacy. The majority of the participants (70.7%) reported low adherence, while 24.1% reported moderate and 5.2% reported high adherence. The results of the Chi-square test showed a significant relationship between age, comorbidities, type of treatment, satisfaction with treatment, the experience of adverse effects, and health literacy with medication adherence (*P* < 0.05 for all). The final constructed model of stepwise multiple linear regression was highly statistically significant. The highest beta coefficient in the final model belonged to the total health literacy score.

Conclusions.

Based on the results, medication adherence among Iranian psoriasis patients is low. Health literacy correlates most strongly with medication adherence and is the best variable to determine it. Improving the access to the internet and the ICTs to enhance the patients` health literacy along with developing the patient education approaches and techniques should be considered by health policymakers.

## Background

Psoriasis is a common chronic inflammatory skin disease [1–3] that affects men and women of all ages [4]. It has recently been recognized by the World Health Organization (WHO) as a serious, painful, disfiguring, and non-communicable disease [5]. Epidemiologicaldata on Psoriasis is so limited worldwide and they are only available in 20 countries, meaning that such data are not available in low- and middle-income countries such as Iran [4]. However, it is estimated that approximately 125 million people worldwide suffer from this disease [2]. The worldwide prevalence of psoriasis in adults, ranges from 0.51% to 11.43%, depending on genetic and immune-mediated components and environmental factors. The incidence increases with age [2–4, 6], making psoriasis a global problem [5]. Based on the severity of the disease, psoriasis is classified as mild, moderate, or severe [3]. Regardless of the severity of the disease, almost 60% of psoriasis patients describe the disease as something that severely affects their quality of life (QOL) [7, 8]. It can also directly impact patients’ work productivity [9]. Psoriasis can manifest itself in many different forms. In addition to the skin, it can involve nails, joints, and other organs as well, and is associated with a number of comorbidities. People suffering from psoriasis are more likely to develop other non-communicable diseases such as psoriatic arthritis, cardiovascular disorders, diabetes, metabolic syndrome, depression, Crohn’s disease, cancer, and nonalcoholic fatty liver disease [4, 10], and experience significant physical, psychological, social, and economic burden depending on the severity and location of the lesions [7, 8, 11], making the cost of psoriasis significant for both patients and healthcare systems [12]. A systematic review estimated the annual cost of psoriasis in the United States at about $112 billion in 2013 [13].

Since the etiology is not yet fully understood, treatment usually focuses on controlling the acute symptoms [4]. A multifaceted treatment approach may be required to manage a complicated disease; however, the complexity of such treatment plans may hinder medication adherence [14]. Unfortunately, adherence is often poor in treating skin diseases such as psoriasis [15, 16], which is the leading cause of failure to control the disease and its exacerbation [8]. The term "adherence" refers to the extent to which a patient’s behavior (including medication use) is consistent with recommendations agreed upon with the healthcare provider [17]. WHO reports show that the medication adherence rate in developing countries is about 50% [18], which is a public health problem because inadequate medication adherence leads to poor treatment outcomes, increased risk of developing comorbidities, inefficient use of healthcare resources, and increased treatment costs [9, 19]. Indeed, poor medication adherence could necessitate stronger and more expensive medications, which could place a significant burden on the healthcare system [20].

On the other hand, individuals with sufficient health literacy and a good understanding of health information are more likely to control their health status. A patient’s ability to seek, process, understand, and use health information is defined as health literacy [20, 21], the lack of which is one of the factors that can explain poor self-management in psoriasis [20]. Accordingly, the importance of health literacy assessment as a factor influencing adherence in psoriasis patients cannot be overstated [20, 21]. However, our in-depth literature review revealed that research on medication adherence and health literacy in psoriasis patients has not been sufficiently explored, rendering more opportunities to study. More specifically, to the best of our knowledge, no previous study has been conducted in Iran to investigate health literacy and medication adherence in psoriasis patients. Given the prevalence rate of psoriasis provided by WHO [5] and the population size of Iran, it is worthwhile to pursue. We believed that the findings of the current study not only can shed the light for health policymakers and healthcare providers for better planning and more applied designing of the interventions to increase the patients’ medication adherence, but they also generate the solid evidence for international practitioners who dealing with psoriasis.

## Methods

### Study design and participants

This is a cross-sectional study among Iranian patients with psoriasis to investigate medication adherence and its relationship with health literacy. As the data were collected during the COVID-19 pandemic, researchers faced severe limitations to contact patients inperson because of the tight rule of social distancing imposed by the government. This forced researchers to find the most suitable alternative method of collecting data. Having searched all available options, the authors finally came up with the online survey for some reasons. First, this significantly helped us to abide by the rules released by the government, leading to keep the safety of both researchers and patients. Second, this further provided us with a great chance to reach patients who were geographically distributed. Third, taking the robustness of results into account, a large portion of patients showed their interest to participate in the study because of deploying a convenient survey tool. Therefore, an online survey link was created and posted on the relevant social media platforms (e.g., Psoriasis Talk website, channels, and groups). This led to a convenience sampling method. Each participant was allowed to submit the questionnaire only once, and all questions had to be answered before the successful final submission of the questionnaire. There were about 60 million people aged above 18 years in the time of the study. Considering the prevalence rate (1.25%) of psoriasis in Iran [22], the size of 750,000 patients has been estimated as a target population. Using the Cochran formula, we need 384 participants for a confidence level of 95%. The survey was conducted between July 26, 2020, and January 5, 2021, and a total of 575 samples were collected, making the results more robust The study included Iranian psoriasis patients between 18 and 67 who agreed voluntarily to participate in the study. They had been on one of the following medical treatments prescribed by a dermatologist for at least one month: topical therapy, oral therapy, injection therapy, and phototherapy.

### Study tools

Study participants were asked to complete a three-part questionnaire. The first part of the questionnaire addressed socio-demographic characteristics (age, gender, education level, marital status, employment status, income, and insurance coverage) and psoriasis characteristics (disease severity, duration of the disease, treatment type, number of current medications, monthly treatment costs, overall satisfaction with treatment, adverse effects of medications, comorbidities, and smoking habits).

The second part of the questionnaire was the eight-item Questionnaire Morisky Medication Adherence Scale (MMAS-8) in Persian, which was a structured and self-report tool, used to assess medication adherence rates. The Persian version of the MMAS has been validated in Iran and is widely used by researchers for chronic diseases such as diabetes [23] and hypertension [24, 25]. The scores of which could be summed between zero and eight using a previously developed method. Each item could further assess adherence rates by measuring a specific medication-taking behavior. In addition, this scale had seven yes/no questions and one item with a five-point Likert scale. Accordingly, the yes and no responses to the dichotomous items were scored as zero and one, respectively, except for item five, which was reverse scored. The possible responses to item eight could also include five possible responses ranging from "always" to "never/ rarely," with "always" and "often" scored as zero, and all other responses scored as one. Finally, the total MMAS-8 score could be calculated by adding all eight individual question scores. Therefore, patients with a score of 8 could reflect a high medication adherence rate, and those with a score of 7 or 6 and lower than 6 could represent moderate and low rates, respectively [26].

Finally, the third part of the questionnaire was the Health Literacy for Iranian Adults (HELIA) tool, developed and validated by Montazeri A for the Iranian population. This questionnaire consists of 33 items and assesses health literacy in five dimensions: access (six items), reading (four items), understanding (seven items), appraisal (four items), and decision (12 items). The total health literacy score was calculated by summing the scores for each item and ranged from 0 to 100. Health literacy was categorized as high with a score between 84.1 and 100, adequate with a score between 50.1 and 84, and insufficient with a score less than 50 [27].

### Data analysis

Statistical analyses were carried out using SPSS software, version 22 (IBM, SPSS Statistics 22). First, descriptive statistics were calculated to summarize the socio-demographic characteristics, medication adherence rates, and health literacy levels (namely scores). In addition, the Kolmogorov–Smirnov test was used to assess the normal distribution of all data. Since the normality assumption was not met, non-parametric tests such as chi-square and Kruskal–Wallis test were applied to compare the categorical and continuous data between the medication adherence and health literacy groups at the significant level of 0.05. In fact, if the null hypothesis is rejected in the Chi-square test, the researcher goes to the Kruskal–Wallis test to determine which group of people had more medication adherence, or in other words, received a better score on the Morisky test. In the Kruskal–Wallis test, the comparison between groups is made by comparing the ranks so that when a group’s mean rank is higher than the other groups, the observation values in that group tend to be higher than those of other groups. Regression analysis is a robust and reliable statistical method of identifying which independent variables impact a dependent variable. Also, the process of performing a regression allows us to confidently determine which factors matter most, which factors can be ignored, and how these factors influence each other; hence, in the current study, Stepwise multiple linear regression was used to assess the influence of independent variables that were significantly associated with medication adherence based on the Chi-square test. The significance level in all statistical tests was considered less than 0.05. Notable results were presented in tables with more detailed numerical values. The Cronbach’s alpha index was used to test reliability, which was 0.71 for the medication adherence questionnaire and 0.93 for the health literacy questionnaire, indicating good internal consistency.

## Results

The demographic data of 575 psoriasis patients are summarized in Table 1. The mean age of the participants was 37.45 years (ranging from 18 to 67), and 52.5% of the participants were female. Of the 575 participants, more than half (54.6%) were treated with topical treatment, 23.9% with injection treatment, 20.3% with oral treatment, and 1.2% with phototherapy. More than half (55.6%) of the participants had experienced adverse effects from the medications. Regarding their overall satisfaction with the treatment, 49.2% of the participants expressed low satisfaction, 38.5% expressed moderate satisfaction, and 12.3% expressed high satisfaction. Other results showed that the mean score of health literacy in the study population was 74.3 ± 14.23, and the mean score of medication adherence was 4.1 ± 2.18.

**Table 1:** Demographic characteristics of Psoriasis patients (*n* = 575)

Variable	Frequency	(Percent %)
Age (years)		
range	37.45*	10.399**
	18–67	
≤ 20	18	(3.1)
21–30	136	(23.6)
31–40	239	(41.6)
41–50	118	(20.6)
51–60	48	(8.3)
60 <	16	(2.8)
Gender		
Female	302	(52.5)
Male	273	(47.5)
Marital status		
Single	163	(28.4)
Married	393	(68.3)
Widowed/Divorced	19	(3.3)
Educational level		
Lower than high school graduate	44	(7.6)
High school graduate	153	(26.7)
Higher than high school graduate	378	(65.7)
Employment status		
Employed	294	(51.1)
Student	40	(6.9)
Housewife	153	(26.7)
Jobless	88	(15.4)
Income (monthly) In dollars		
< 500	260	(45.2)
500–1000	167	(29.1)
1000–1500	81	(14.2)
1500 <	67	(11.6)
Insurance coverage		
Insured	420	(73.0)
Non-insured	155	(27.0)
Smoking		
Smoker	106	(18.4)
Non-smoker	469	(81.6)
Comorbidity		
Yes	190	(33.1)
No	385	(66.9)
Years with Psoriasis		
< 2	39	(6.9)
2–5	79	(13.7)
5–10	87	(15.1)
10 <	370	(64.3)
Psoriasis severity		
Mild	105	(18.2)
Moderate	295	(51.3)
Severe	175	(30.5)
Treatment type		
Topical	314	(54.6)
Injection	137	(23.9)
Oral	117	(20.3)
Phototherapy	7	(1.2)
Number of current medications		
1–3	482	(83.9)
3 <	93	(16.1)
Treatment cost (monthly) In dollars		
< 25	201	(35.0)
25–50	105	(18.2)
50–75	78	(13.5)
75 <	191	(33.3)
Experience of adverse effects by medication		
Yes	320	(55.6)
No	255	(44.4)
Overall satisfaction with treatment		
Low	283	(49.2)
Medium	221	(38.5)
High	71	(12.3)

Mean (*). Standard deviation (**)

Descriptive statistics of health literacy are given in Table 2. As table shows, the lowest and highest score of health literacy were observed in the decision and understanding dimensions, respectively.

**Table 2:** Descriptive Statistics of Health Literacy Dimensions (n = 575)

Name of dimension	Number of items	Mean score	Std. Deviation
Access	6	71.5	19.31
Reading	4	76.7	20.35
Understanding	7	84.5	14.50
Appraisal	4	74.4	19.06
Decision	12	64.4	17.59

As shown in Table 3, out of the total participants, 28.8% had high health literacy, 67.1% had adequate health literacy, and 4% had inadequate health literacy. The results of chi-square and Kruskal–Wallis tests showed that health literacy was significantly related to the duration of psoriasis (*P* < 0.05), employment status (*P* < 0.05), treatment type (*P* < 0.05), and satisfaction with treatment (*P* < 0.05) so that the mean rank score of health literacy was higher in patients with a history of psoriasis of more than 10 years and less than 2 years than other patients. As for employment status, the highest and lowest mean rank scores of health literacy were observed in students and unemployed patients. Regarding the treatment type, the mean rank value of health literacy was highest in patients who received phototherapy, followed by patients who used injection therapy, and then by patients who received topical treatment. Moreover, the lowest mean rank score of health literacy was observed in patients who received oral treatment. Patient satisfaction with treatment was statistically associated with a high mean health literacy score (*P* < 0.05), which means that as satisfaction with treatment increased, so did the mean rank score of health literacy.

**Table 3:** Comparison of Health Literacy Mean Rank Score in Relation to associated variables among Psoriasis Patients (*n* = 575)

Variable	Health literacy level (%)				Health literacy Mean Rank Score
	Insufficient (4.0)	Sufficient (67.1)	High (28.8)	*P*-value	
Age (years)					
≤ 20	(7.7)	(69.2)	(23.1)	0.349	
21–30	(7.0)	(62.0)	(31.0)		
31–40	(4.0)	(71.0)	(25.0)		
41–50	(0.0)	(69.0)	(31.0)		
51–60	(2.9)	(57.1)	(40.0)		
60 <	(8.3)	(66.7)	(25.0)		
Gender					
Female	(3.6)	(64.4)	(32.0)	0.314	
Male	(4.5)	(70.1)	(25.4)		
Marital status					
Single	(5.8)	(73.3)	(20.8)	0.121^*^	
Married	(3.5)	(64.0)	(32.5)		
Widowed/Divorced	(0.0)	(78.6)	(21.4)		
Educational level					
Lower than high school graduate	(9.4)	(71.9)	(18.8)	0.157	
High school graduate	(6.2)	(64.6)	(29.2)		
Higher than high school graduate	(2.5)	(67.6)	(29.9)		
Employment status					
Employed	(2.3)	(68.1)	(29.6)	0.044^*^	217.24
Student	(3.4)	(58.6)	(37.9)		224.91
Housewife	(5.3)	(61.9)	(32.7)		217.32
Jobless	(7.7)	(76.9)	(15.4)		179.58
Income (monthly) In dollars					
< 500	(5.8)	(69.1)	(25.1)	0.134^*^	
500–1000	(1.6)	(70.7)	(27.6)		
1000–1500	(1.7)	(63.3)	(35.0)		
1500 <	(6.1)	(55.1)	(38.8)		
Insurance coverage					
Insured	(3.9)	(64.7)	(31.4)	0.163	
Non-insured	(4.4)	(73.7)	(21.9)		
Smoking					
Smoker	(5.1)	(70.5)	(24.4)	0.571	
Non-smoker	(3.8)	(66.4)	(29.9)		
Comorbidity					
Yes	(2.9)	(68.6)	(28.6)	0.680	
No	(4.6)	(66.4)	(29.0)		
Years with Psoriasis					
< 2	(6.9)	(65.5)	(27.6)	0.046^*^	206.90
2–5	(5.2)	(70.7)	(24.1)		205.75
5–10	(10.9)	(60.9)	(28.1)		198.20
10 <	(1.8)	(68.0)	(30.1)		217.12
Psoriasis severity					
Mild	(1.3)	(71.4)	(27.3)	0.225	
Moderate	(3.2)	(68.7)	(28.1)		
Severe	(7.0)	(62.0)	(31.0)		
Treatment type					
Topical	(3.5)	(72.7)	(23.8)	0.037^*^	204.06
Injection	(4.0)	(54.5)	(41.6)		234.71
Oral	(5.8)	(67.4)	(26.7)		203.37
Phototherapy	(0.0)	(60.0)	(40.0)		268.40
Number of current medications					
1–3	(3.7)	(67.9)	(28.5)	0.606	
3 <	(5.9)	(63.2)	(30.9)		
Treatment cost (monthly) In dollars					
< 25	(3.4)	(67.6)	(29.1)	0.366	
25–50	(1.3)	(74.0)	(24.7)		
50–75	(7.0)	(56.1)	(36.8)		
75 <	(5.0)	(67.4)	(27.7)		
Experience of adverse effects by medication					
Yes	(4.7)	(67.2)	(28.1)	0.711	
No	(3.2)	(67.0)	(29.8)		
Overall satisfaction with treatment					
Low	(5.8)	(69.2)	(25.0)	0.000	201.05
Medium	(2.5)	(71.8)	(25.8)		206.03
High	(1.9)	(44.2)	(53.8)		274.51
Medication adherence level					
Low	(4.7)	(71.2)	(21.4)	0.015	196.01
Medium	(2.9)	(57.8)	(39.2)		242.51
High	(0.0)	(54.5)	(45.5)		287.89
Cronbach’s alpha of health literacy = 0.93					

*Fisher’s exact test

Based on the results of MMAS-8, 57.6% of the participants mentioned forgetting to take medicines as one of the reasons for non-adherence. 54.6% of the participants stopped taking their medication without telling their doctor just because they felt worse when they took it. And,78.3% of participants felt hassled and found it difficult to stick to their treatment plan.

As in Table 4 shows, the majority of the participants (70.7%) reported low adherence, while 24.1% reported moderate and 5.2% reported high adherence. According to the results of the chi-square and Kruskal–Wallis tests, age, comorbidities, treatment type, experience of adverse effects, satisfaction with treatment, and health literacy were significantly associated with medication adherence (*P* < 0.05). High levels of adherence were observed in older participants (except in the age group of 31 to 40 years and over 60 years). Participants taking medications for comorbidities had a higher mean rank value of medication adherence compared with participants without comorbidities. Medication adherence was statistically higher in patients who used oral or injection treatment than in those who used topical treatment or phototherapy (*p* < 0.05). In addition, significantly better adherence was observed in patients with higher satisfaction with treatment and experienced no adverse effects from the medications. A comparison of mean rank score of medication adherence in relation to the level of health literacy of the study participants showed better adherence among those with higher levels of health literacy. Consequently, the lowest and highest mean rank score of medication adherence were observed in participants with inadequate and high health literacy levels, respectively.

**Table 4:** Comparison of Adherence Mean Rank score in relation to associated variables among Psoriasis patients (*n* = 575)

Variable	Medication adherence level (%)				Adherence Mean Rank Score
	Low (70.7)	Medium (24.1)	High (5.2)	*P*-value	
Age (years)					
≤ 20	(53.8)	(38.5)	(7.7)	0.004	201.77
21–30	(75.0)	(19.0)	(6.0)		209.42
31–40	(77.8)	(18.2)	(4.0)		190.32
41–50	(66.7)	(28.7)	(4.6)		226.67
51–60	(40.0)	(48.6)	(11.4)		288.56
60 <	(66.7)	(33.3)	(0.0)		232.92
Gender					
Female	(68.9)	(25.2)	(5.9)	0.660	
Male	(72.6)	(22.9)	(4.5)		
Marital status					
Single	(69.2)	(25.0)	(5.8)	0.161	
Married	(70.6)	(24.9)	(4.5)		
Widowed/Divorced	(85.7)	(0.0)	(14.3)		
Educational level					
Lower than high school graduate	(62.5)	(37.5)	(0.0)	0.238	
High school graduate	(69.0)	(23.9)	(7.1)		
Higher than high school graduate	(72.3)	(22.7)	(5.0)		
Employment status					
Employed	(72.7)	(21.3)	(6.0)	0.530	
Student	(65.5)	(27.6)	(6.9)		
Housewife	(67.3)	(30.1)	(2.7)		
Jobless	(72.3)	(21.5)	(6.2)		
Income (monthly) In dollars					
< 500	(72.8)	(23.6)	(3.7)	0.798	
500–1000	(69.9)	(22.8)	(7.3)		
1000–1500	(68.3)	(25.0)	(6.7)		
1500 <	(67.3)	(28.6)	(4.1)		
Insurance coverage					
Insured	(69.9)	(24.9)	(5.2)	0.815	
Non-insured	(72.8)	(21.9)	(5.3)		
Smoking					
Smoker	(75.6)	(21.8)	(2.6)	0.402	
Non-smoker	(69.6)	(24.6)	(5.8)		
Comorbidity					
Yes	(65.7)	(31.4)	(2.9)	0.023	213.21
No	(73.1)	(20.5)	(6.4)		211.40
Years with Psoriasis					
< 2	(65.5)	(27.6)	(6.9)	0.213^*^	
2–5	(63.8)	(25.9)	(10.3)		
5–10	(73.4)	(18.8)	(7.8)		
10 <	(72.1)	(24.6)	(3.3)		
Psoriasis severity					
Mild	(68.8)	(26.0)	(5.2)	0.684	
Moderate	(73.3)	(21.2)	(5.1)		
Severe	(66.7)	(27.9)	(5.4)		
Treatment type					
Topical	(82.7)	(15.2)	(2.2)	0.000^*^	175.56
Injection	(53.5)	(36.6)	(9.9)		263.37
Oral	(58.1)	(33.7)	(8.1)		253.41
Phototherapy	(80.0)	(20.0)	(0.0)		145.60
Number of current medications					
1–3	(72.1)	(22.3)	(5.6)	0.100	
3 <	(63.2)	(33.8)	(2.9)		
Treatment cost (monthly) In dollars					
< 25	(75.7)	(19.6)	(4.7)	0.381	
25–50	(75.3)	(22.1)	(2.6)		
50–75	(66.7)	(26.3)	(7.0)		
75 <	(64.5)	(29.1)	(6.4)		
Experience of adverse effects by medication					
Yes	(75.3)	(21.7)	(3.0)	0.019	202.49
No	(64.9)	(21.1)	(8.0)		223.89
Overall satisfaction with treatment					
Low	(76.0)	(20.7)	(3.4)	0.001	188.30
Medium	(71.8)	(22.7)	(5.5)		218.04
High	(46.2)	(42.3)	(11.5)		287.86
Health literacy level					
Insufficient	(82.4)	(17.6)	(0.0)	0.015	153.12
Sufficient	(75.0)	(20.8)	(4.2)		196.92
High	(59.0)	(32.8)	(8.2)		255.32
Cronbach’s alpha of adherence = 0.71					

*Fisher’s exact test

According to Table 5, stepwise multiple linear regression revealed a statistically significant association between the study variables such as age, satisfaction with treatment, health literacy, and medication adherence. The final constructed model with these variables was highly statistically significant (*F* = 24.171, *p* < 0.0001, *R*^2^ = 0.148). The highest beta coefficient in the third step (0.249) belonged to the total score of health literacy; therefore, it can be said that health literacy had the highest correlation with medication adherence and was the best variable to determine medication adherence. After health literacy, satisfaction with treatment (β = 0.212), and age (β = 0.139) were identified as most influential factors.

**Table 5:** The final constructed model of Stepwise Multiple Linear Regression Analysis (*n* = 575)

Model	Independent Variable	R	R^2^	Adjusted R^2^	F (P)	Unstandardized Coefficients		Standardized Coefficients	t (Sig)
						B	Std. Error	β	
3	Total health literacy score	0.384	0.148	0.141	24.171 (0.000)	0.038	0.007	0.249	5.446 (0.000)
	Overall satisfaction with treatment					0.667	0.144	0.212	4.636 (0.000)
	Age					0.029	0.009	0.139	3.083 (0.002)
Durbin-Watson = 2.044									

Dependent variable: Adherence total score

## Discussion

Recent studies have highlighted the low adherence rate in patients with chronic dermatological conditions such as psoriasis [14, 28–31]. The results of the current study also confirmed that low medication adherence is common among Iranian patients with psoriasis. A study by Alsubeeh. et al. (2019) [30] showed that psoriasis patients had the lowest mean adherence scores in Saudi Arabia compared to other patients with chronic skin diseases. This could be since the experience of medication efficacy and overall satisfaction with treatment is lower in psoriasis patients compared to patients with other skin diseases [31]. A systematic review by Thorneloe et al. (2013) [16] reported low rates of medication adherence in patients with psoriasis regardless of the treatment type, disease severity, or type of adherence measurement tool used. Based on a WHO report [5], low adherence is due in part to inadequate communication between healthcare providers and patients regarding medication instructions, misperception of possible adverse effects, and mistaken expectations regarding the rate and extent of improvement.

As expected, high adherence was observed among those satisfied with their treatment and experienced no adverse effects from medications, which is consistent with many previous studies conducted on psoriasis patients [5, 17, 21, 30–32]. Thus, if physicians and pharmacists spend sufficient time explaining to patients the condition, the need for medication, expectations of treatment, and possible adverse effects, and if they address patients’ concerns and clear up any misconceptions about the proposed treatments, medication adherence rates are likely to increase [5, 14].

In line with several studies that have highlighted the influence of various factors on the medication adherence rate of psoriasis patients [21, 29, 30, 32], this study also showed that medication adherence was influenced by several factors such as age, treatment type, satisfaction with treatment, experience of adverse effects, and health literacy. Of all these variables, health literacy is correlated most strongly with medication adherence and was the best variable to describe it. Moreover, the positive association between health literacy and medication adherence indicated that patients with higher overall health literacy had better medication adherence. Ostini. et al. (2014) [33] reported that patients with an average level of health literacy had higher adherence than patients with below and above average health literacy. A study by Rasmussen GS. et al. [34] indicated that psoriasis patients with lower levels of health literacy might have difficulty adhering to complex treatment modalities. In contrast to Larsen et al. (2019) [20], who reported that a large number of psoriasis patients had shortcomings in health literacy, the majority of the participants in the present study had high and sufficient levels of health literacy in so many related areas, which could be attributed to the online nature of the survey. Since no significant relationship was found between educational status and health literacy, it can be inferred that patients who use social media and the Internet have higher health literacy. Increasing the access to the internet and the Information Communication Technologies (ICTs) can be among the approaches of the government at a macro level to help improving the health literacy of patients. A high level of health literacy among a significant proportion of the Iranian population with psoriasis is a positive factor for psoriasis control, as people with chronic diseases are increasingly expected to take charge of their own health. Since limited health literacy is associated with poorer patient-physician communication, health-related skills, health outcomes, and treatment adherence [21], high levels of health literacy may be a helpful strategy to improve self-management skills, treatment adherence, and quality of life [20].

Better adherence was also observed in older patients. This finding is consistent with a study by Saeki. et al. (2015) [31], which showed significantly better adherence in older patients when they received oral medications. In contrast, Ahn. et al. (2017) [17] reported that higher adherence was observed among psoriasis patients in younger patients That can be justified because of the cultural differences.

Several studies show that adherence is significantly higher with oral treatment than with topical treatment [28, 30, 31]. The present study also confirms this result. In addition, it was found that patients who received injectable treatment had the highest adherence compared to other psoriasis treatment types; it was followed by adherence to oral treatment, adherence to topical treatment, and adherence to phototherapy, respectively. Injectable medications are often preferred over orally administered medications. This may contribute to increased adherence to injectable medications compared to oral medications. Positive feedback on adherence due to the high efficacy of injection treatment in psoriasis patients may also contribute to long-term adherence to these medications. Patients treated with injection biologics also tend to suffer from a more severe form of psoriasis than patients using topical treatment or phototherapy. Theoretically, this could lead to higher motivation and, therefore, better medication adherence in those receiving injectables. Common reasons for poor adherence to topical treatments include low efficacy, the increased time required for application, and poor cosmetic properties of the preparation used for psoriasis treatment (given the presence of greasy, sticky, or smelly carriers) [14]. In a study by Saeki. et al. (2015) [31], it was found that although the efficacy of topical medication was relatively high, topical adherence and treatment satisfaction was low, suggesting that a large number of psoriasis patients have locally refractory lesions that cause low adherence and satisfaction.

In sum, it is recommended to evaluate the influence of other factors associated with medication adherence such as quality of life and psychological well-being on its level. The results of such a study should be considered in treatment decision-making [1] to help providers successfully engage patients in treatment and get them to adhere to it by identifying and addressing common barriers to adherence. Better clinical outcomes and lower healthcare costs can thus be conveniently achieved by improving patient adherence [21].

### Limitations

Like other studies, this study may have some limitations. First, the selection bias of online survey that caused missing of data on patients with limited access to the internet or social media and illiterate patients, resulting in less generalizability of results. Second limitation is concerned with self-report bias in terms of recall and social desirability. To avoid this type of bias, it is recommended to measure adherence and health literacy using direct methods. Also, for confirming the clinical manifestation of the participants, obtaining the data from the medical records is recommended.

### Strengths

To the best of our knowledge, the present study is the first of its kind to investigate the relationship between health literacy and medication adherence in Iranian psoriasis patients. Compared to other cross-sectional studies in the literature, the large sample size and the use of comprehensive and valid measurement tools, namely MMAS-8 and HELIA, are among the salient features of this study. In addition, several patient-related factors were assessed to predict possible associations with medication adherence in psoriasis patients, and the importance of health literacy to assess medication adherence was highlighted.

## Conclusions

Based on our analysis, medication adherence among Iranian psoriasis patients was low. Moreover, among all factors examined in this study, age, satisfaction with treatment, and health literacy were positive predictors of adherence. Approximately 14.8% of the changes in adherence scores are explained by the three variables listed above. Of all these variables, health literacy predicts medication adherence with much power and is the best variable to determine it. Improving patients` health literacy through compelling strategies should be seriously implemented. To this end, all parties from government to health professional should contribute.

## Data Availability

Data is available upon reasonable request by the corresponding author.

## Abbreviations

COVID-19: Coronavirus Disease of 2019
e.g.: (Exempli gratia) for example
HELIA: Health Literacy for Iranian Adults
ICTs: Information Communication Technologies
MMAS-8: Morisky Medication Adherence Scale-8
QOL: Quality of life
WHO: World Health Organization

## References

[1] Belinchón I, Rivera R, Blanch C, Comellas M, Lizán L (2016). Adherence, satisfaction and preferences for treatment in patients with psoriasis in the European Union: a systematic review of the literature. Patient Prefer Adherence.

[2] Takeshita J, Grewal S, Langan SM, Mehta NN, Ogdie A, Van Voorhees AS (2017). Psoriasis and comorbid diseases: epidemiology. J Am Acad Dermatol.

[3] Augustin M, Alvaro-Gracia JM, Bagot M, Hillmann O, van de Kerkhof PC, Kobelt G (2012). A framework for improving the quality of care for people with psoriasis. J Eur Acad Dermatol Venereol.

[4] Michalek IM, Loring B, John SM (2017). A systematic review of worldwide epidemiology of psoriasis. J Eur Acad Dermatol Venereol.

[5] World Health O (2016). Global report on psoriasis.

[6] Strober BE, van der Walt JM, Armstrong AW, Bourcier M, Carvalho AVE, Chouela E (2019). Clinical goals and barriers to effective psoriasis care. Dermatol Ther.

[7] Kwan Z, Bong YB, Tan LL, Lim SX, Yong ASW, Ch'ng CC (2018). Determinants of quality of life and psychological status in adults with psoriasis. Arch Dermatol Res.

[8] Armstrong AW, Schupp C, Wu J, Bebo B (2012). Quality of life and work productivity impairment among psoriasis patients: findings from the National Psoriasis Foundation survey data 2003–2011. PLoS ONE.

[9] Zschocke I, Mrowietz U, Karakasili E, Reich K (2014). Non-adherence and measures to improve adherence in the topical treatment of psoriasis. J Eur Acad Dermatol Venereol.

[10] Boehncke WH, Schön MP (2015). Psoriasis. Lancet.

[11] NajafiGhezeljeh TSK, Hoseini A-F (2018). The effect of self-management education on the quality of life and severity of the disease in patients with severe psoriasis: a non-randomized clinical trial. NPT.

[12] Parisi R, Symmons DP, Griffiths CE, Ashcroft DM (2013). Global epidemiology of psoriasis: a systematic review of incidence and prevalence. J Invest Dermatol.

[13] Brezinski EA, Dhillon JS, Armstrong AW (2015). Economic burden of psoriasis in the United States: a systematic review. JAMA Dermatol.

[14] Haidari W, Quan EY, Cline A, Feldman SR, Feldman SR, Cline A, Pona A, Kolli SS (2020). Adherence in Psoriasis. Treatment adherence in dermatology.

[15] Feldman SR, Vrijens B, Gieler U, Piaserico S, Puig L, van de Kerkhof P (2017). Treatment adherence intervention studies in dermatology and guidance on how to support adherence. Am J Clin Dermatol.

[16] Thorneloe RJ, Bundy C, Griffiths CE, Ashcroft DM, Cordingley L (2013). Adherence to medication in patients with psoriasis: a systematic literature review. Br J Dermatol.

[17] Ahn CS, Culp L, Huang WW, Davis SA, Feldman SR (2017). Adherence in dermatology. J Dermatolog Treat.

[18] World Health O (2003). Adherence to long-term therapies : evidence for action / [edited by Eduardo Sabaté].

[19] Lam WY, Fresco P (2015). Medication Adherence measures: an overview. Biomed Res Int.

[20] Larsen MH, Strumse YAS, Borge CR, Osborne R, Andersen MH, Wahl AK (2019). Health literacy: a new piece of the puzzle in psoriasis care? A cross-sectional study. Br J Dermatol.

[21] Pona A, Cline A, Feldman SR, Feldman SR, Cline A, Pona A, Kolli SS (2020). Reasons for Nonadherence. Treatment adherence in dermatology.

[22] Zarei E, Gholamhosseini A, Ghandi N (2021). Estimation of direct and indirect costs of one year treatment for psoriasis outpatients in Iran: a study in Razi Hospital in 2017–2018. Dermatol Cosmet.

[23] Laghousi D, Rezaie F, Alizadeh M, Asghari JM (2021). The eight-item Morisky medication adherence scale: validation of its Persian version in diabetic adults. Caspian J Intern Med.

[24] Behnood-Rod A, Rabbanifar O, Pourzargar P, Rai A, Saadat Z, Saadat H (2016). Adherence to antihypertensive medications in iranian patients. Int J Hypertens.

[25] Moharamzad Y, Saadat H, NakhjavanShahraki B, Rai A, Saadat Z, Aerab-Sheibani H (2015). Validation of the Persian version of the 8-Item Morisky Medication Adherence Scale (MMAS-8) in Iranian hypertensive patients. Glob J Health Sci.

[26] Morisky DE, Ang A, Krousel-Wood M, Ward HJ (2008). Predictive validity of a medication adherence measure in an outpatient setting. J Clin Hypertens.

[27] Montazeri AL, Tavousi M, Rakhshani FA, Azin SA, Jahangiri K, Ebadi M (2014). Health Literacy for Iranian Adults (HELIA): development and psychometric properties. Health Mon J Iran Inst Health Sci Res.

[28] Wang Q, Luo Y, Lv C, Zheng X, Zhu W, Chen X (2020). Nonadherence to treatment and patient-reported outcomes of psoriasis during the COVID-19 epidemic: a web-based survey. Patient Prefer Adherence.

[29] Wang W, Qiu Y, Zhao F, Zhang F (2019). Poor medication adherence in patients with psoriasis and a successful intervention. J Dermatolog Treat.

[30] Alsubeeh NA, Alsharafi AA, Ahamed SS, Alajlan A (2019). Treatment adherence among patients with five dermatological diseases and four treatment types - a cross-sectional study. Patient Prefer Adherence.

[31] Saeki H, Imafuku S, Abe M, Shintani Y, Onozuka D, Hagihara A (2015). Poor adherence to medication as assessed by the Morisky Medication Adherence Scale-8 and low satisfaction with treatment in 237 psoriasis patients. J Dermatol.

[32] Svendsen MT, Andersen F, Hansen J, Johannessen H, Andersen KE (2017). Medical adherence to topical corticosteroid preparations prescribed for psoriasis: a systematic review. J Dermatolog Treat.

[33] Ostini R, Kairuz T (2014). Investigating the association between health literacy and non-adherence. Int J Clin Pharm.

[34] Rasmussen GS, Maindal HT, Lomborg K (2012). Self-management in daily life with psoriasis: an integrative review of patient needs for structured education. Nutr Res Pract.

